# TWEAK Promotes the Proliferation of Squamous Cell Carcinoma Cells Through Activating cIAP1 Signals

**DOI:** 10.3389/fonc.2020.00439

**Published:** 2020-04-15

**Authors:** Lili Liang, Chuantao Cheng, Guanglei Hu, Xuening Wang, Jing Liu, Zhu Yan, Weihui Zeng, Yumin Xia

**Affiliations:** ^1^Department of Dermatology, The Second Affiliated Hospital of Xi'an Jiaotong University, Xi'an, China; ^2^Department of Dermatology, The Affiliated Shanxi Provincial People's Hospital of Shanxi Medical University, Taiyuan, China

**Keywords:** cIAP1, Fn14, proliferation, squamous cell carcinoma, TWEAK

## Abstract

Recent studies showed that tumor necrosis factor (TNF)-like weak inducer of apoptosis (TWEAK) induces the proliferation of squamous cell carcinoma (SCC) cells. However, the precise mechanism underlying such effect of TWEAK remains unclear. This study was designed to elucidate the role of cellular inhibitor of apoptosis 1 (cIAP1) in TWEAK-induced proliferation of SCC cells. Human SCC cells (SCC-13, A431, and SCC-9) were cultured *in vitro*, receiving the stimulation of TWEAK or TNF-related apoptosis-inducing ligand (TRAIL). We found that TWEAK induced cytoplasmic cIAP1 importation and RIP1 ubiquitination in cells, followed by the activation of canonical nuclear factor kappa B signals. MV1, a cIAP1 inhibitor, abrogated TWEAK-induced proliferation of these cells. Moreover, the interaction between TWEAK and its receptor, fibroblast growth factor-inducible 14 (Fn14), enhanced the expression of TRAIL receptor types 3 and 4 (TRAIL-R3/4). Furthermore, the transfection of TRAIL-R3/4 siRNA abrogated the promotion effect of TWEAK on SCC-13 cell proliferation and cIAP1 expression. Therefore, TWEAK/Fn14 interaction promotes the proliferation of SCC cells through activating cIAP1 signals. Targeting the downstream cIAP1 signals might attenuate the effect of TWEAK on SCC cells.

## Introduction

Tumor necrosis factor (TNF)-related weak inducer of apoptosis (TWEAK) is a soluble cytokine and belongs to the members of the TNF superfamily. TWEAK acts through engaging its cognate receptor, fibroblast growth factor-inducible 14 (Fn14). During recent years, many studies demonstrated that the TWEAK/Fn14 signaling pathway participates in the progression and metastasis of cancers (1–3). TWEAK is mainly synthesized by infiltrating inflammatory cells including macrophages, which also accumulate in cancer tissues (4). Both TWEAK and Fn14 are overexpressed in various malignancies though they have very low levels in normal tissues (2). Moreover, Fn14 is significantly upregulated in cancer cells *in vitro* (5). Currently, TWEAK/Fn14 signals are believed to contribute to the development of cancers through enhancing the proliferation, invasion and migration of cancer cells as well as the angiogenesis, inflammatory responses, and morphogenesis of non-cancer cells (2). Targeting of the TWEAK/Fn14 molecules may prevent the progression of cancers and prolong the survival of murine cancer model (5, 6).

Recently, we found that both TWEAK and Fn14 are highly expressed in human cutaneous squamous cell carcinoma (SCC), and TWEAK/Fn14 interaction promotes the proliferation, migration, and invasion of cultured SCC cell lines (7). Inhibition of Fn14 suppresses the growth of SCC xenografts in nude mice (7). The effect of TWEAK on the fate (proliferation or apoptosis) of keratinocytes is associated with the expression profile of TNF receptor (TNFR) (8). TWEAK/Fn14 interaction induces apoptosis of normal keratinocytes that predominantly express TNFR1. Conversely, TWEAK increases the proliferation of and cytoplasmic import of cellular inhibitor of apoptosis 1 (cIAP1) in TNFR2-overexpressing keratinocytes (8). TWEAK regulates cell fates involving the function of Fn14-TNFR-associated factor-TNFR axis (8, 9). Meanwhile, the TWEAK-Fn14-cIAP1-nuclear factor kappa B (NF-κB) signaling axis is critical in the regulation of myogenesis and muscle homeostasis (10). These findings strongly indicated that cIAP1 may participate in the effect of TWEAK on proliferation of cells including SCC cells.

In fact, receptor-interacting serine/threonine-protein kinase 1 (RIP1) is crucial for the regulation of cell fates, which is associated with TNF signaling (11). As an ubiquitin E3 ligase, cIAP1 exert regulatory functions on RIP1 ubiquitination. However, the precise relationship between the cIAP1 and TWEAK/Fn14 signals remains unclear in malignancies. The purpose of this study was to elucidate the role of cIAP1 in TWEAK-induced proliferation of SCC cells.

## Materials and Methods

### Cell Culture

As described previously, human primary keratinocytes were isolated from foreskin epidermis (12). Human squamous cell carcinoma cells (SCC-13, A431, and SCC-9) were cultured in Dulbecco's modified Eagle's media, which was supplemented with 10% fetal bovine serum (Life Technologies, Carlsbad, CA, USA). Prior to stimulation assays, cells were starved for 24 h in serum-free media. Recombinant human TWEAK (0 to 1000 ng/ml; R&D Systems, Minneapolis, MN, USA), TRAIL (20 ng/ml) or MV1 (1 μM; C_31_H_40_N_4_O_5_; RaystarBio Inc., Hangzhou, China) was administrated to the cultures in some experiments.

### siRNA Transfection

SCC-13 cells were grown in six-well plates. siRNA transfection was performed as described previously (13). The control siRNA oligonucleotide (or that of target molecules) and Lipofectamine 2000 transfection reagent (Life Technologies) were mixed at a ratio of 75 pmol : 7.5 μl, and then added to the cultures for 48 h. The catalog number were #135142 (Fn14 siRNA), #111378 (TRAIL-R3 siRNA), #111376 (TRAIL-R4 siRNA), and Silencer® Control #1 (negative control siRNA). Quantitative real-time polymerase chain reaction (qRT-PCR) and Western blotting were performed to verify the efficiency of transfection (Figure S1).

### qRT-PCR

By using a Trizol reagent (Life Technologies), total RNA was extracted from cell cultures. Then, reverse transcription was performed with a commercial cDNA kit (Invitrogen, Grand Island, NY, USA). SYBR Green Master Mixes (Invitrogen) was used as a fluorescent dye. Then, qRT-PCR was carried out on One-Step PCR System (Applied Biosystems, Carlsbad, CA, USA). All primer sequences (Sangon Inc., Shanghai, China) are listed in Table S1.

### Western Blotting

RIPA lysis buffer (HEART Biotech, Xi'an, China) was used for the extraction of total proteins from cell cultures. The denatured samples were separated on electrophoresis gels, and then transferred onto polyvinylidene difluoride membrane (Millipore, Billerica, MA, USA). Primary antibodies were rabbit IgG that targeted human Fn14, p65, cIAP1, IκBα, phosphorylated IκBα, TNF receptors, TRAIL receptors, or β-actin (1 μg/ml; Cell Signaling). Horseradish peroxidase-conjugated goat anti-rabbit IgG (0.5 μg/ml; Southern Biotech, Birmingham, AL, USA) was the secondary antibody. Finally, signal was developed by using a chemiluminescent kit (Millipore). ImageJ software (National Institutes of Health, Bethesda, MD, USA) was used for the measurement of band intensities. The values of target proteins were normalized to β-actin accordingly.

In some experiments, the protein lysates were subjected to immunoprecipitation with rabbit anti-RIP1 IgG (Abcam, Cambridge, MA, USA) and then probed with anti-ubiquitin IgG (1 μg/ml; Abcam). Immunoprecipitation was performed as described previously (8). Ubiquitinated RIP1 was then detected by Western blotting.

### Immunofluorescence

SCC cells grown on a glass-bottom culture dish (Life Technologies) were fixed in 4% paraformaldehyde solution. Primary antibody was rabbit anti-cIAP1 IgG (2 μg/ml; Abcam). Alexa Fluor 488-labeled goat anti-rabbit IgG was the secondary antibody (2 μg/ml; Abcam). After counterstain with 4',6-diamidino-2-phenylindole (DAPI), cells were observed under a digital confocal microscopy (Leica Company, Wetzlar, Germany).

### Proliferation and Apoptosis Assays

Proliferative cells were visualized by fluorescent staining for Ki67. Alexa Fluor 488-conjugated rabbit anti-Ki67 IgG was used as primary antibody (2 μg/ml; Abcam). A terminal deoxynucleotidyl transferase-mediated dUTP nick end labeling kit (EMD Millipore, Billerica, MA, USA) was used for the detection of apoptotic cells. The percentages of proliferative or apoptotic cells were calculated, respectively.

### Statistical Analysis

Data were described as means ± standard error. The Prism software version 6.0 (GraphPad, La Jolla, CA, USA) was used for statistical analysis. To compare more than two groups, one-way analysis of variance (ANOVA) was first performed. A two-tailed Student's *t*-test was then carried out for comparing the two groups. Significant difference was considered as a *p* value was less than 0.05.

## Results

### TWEAK Induces Cytoplasmic cIAP1 Importation and RIP1 Ubiquitination in SCC Cells

**Figure 1:**
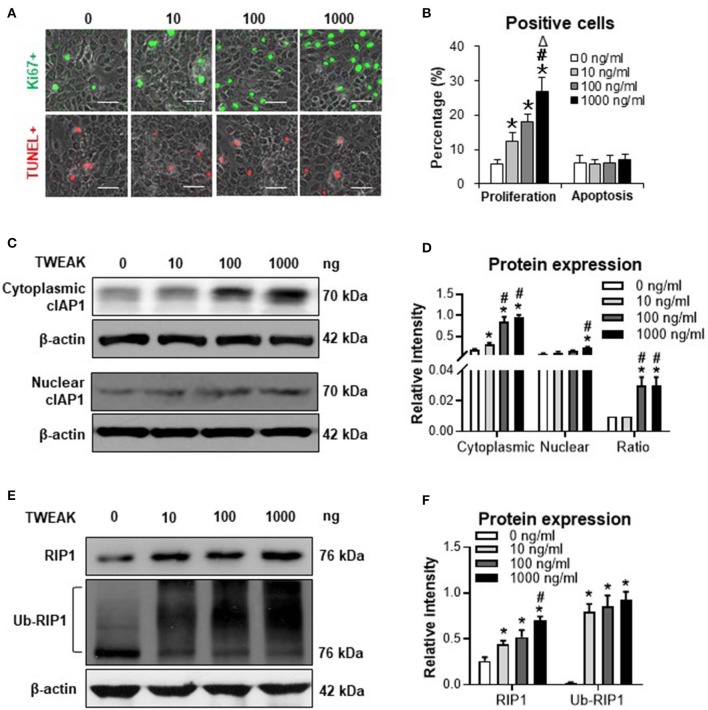
The effect of TWEAK on cIAP1 distribution and RIP1 ubiquitination in SCC-13 cells. SCC-13 cells were cultured *in vitro*, and received 48-h stimulation of TWEAK (0-1000 ng/ml). **(A)** The Ki67- (green) or TUNEL-positive (red) cells were detected by immunofluorescence. **(B)** The percentages of positive cells were calculated accordingly. **(C,D)** By Western blotting, the protein of cIAP1 was detected in cytoplasmic or nuclear fractions. The intensities of Western blot bands were measured with ImageJ software. **(E,F)** By Western blotting, the total or ubiquitinated RIP1 was detected in cell lysates. The band intensities were measured accordingly. Data were obtained from three independent experiments. Representative images are shown. Bar = 20 μm. *p < 0.05, compared with the 0 ng/ml group; #*p* < 0.05, compared with the 10 ng/ml group. Δ*p* < 0.05, compared with the 100 ng/ml group.

Previously, we demonstrated that TWEAK induces proliferation of A431 and SCC-9 cells (SCC cell lines) (7). In this study, SCC-13 cells were utilized to investigate the effect of TWEAK on cell proliferation or apoptosis. It showed that TNFR2 is highly expressed in SCC-13 cells (Figure S2). Moreover, TWEAK promoted proliferation of SCC-13 cells in a dose-dependent manner (0–1000 ng/ml) (Figures 1A,B). However, the apoptotic ratio of cells was not affected upon TWEAK stimulation (Figures 1A,B).

The expression of cIAP1 was determined in cytoplasmic and nuclear extracts. It showed that both cytoplasmic and nuclear cIAP1 protein was upregulated in SCC-13 cells with the addition of TWEAK (Figures 1C,D). Interestingly, the ratio of cytoplasmic to nuclear cIAP1 increased significantly at the dose range of 100 to 1000 ng/ml (Figure 1D). Moreover, the protein expression level of RIP1 was promoted upon TWEAK stimulation, accompanied by the ubiquitination of RIP1 (Figures 1E,F). Similarly, TWEAK also induced cIAP1 expression in A431 and SCC-9 cells (Figure S3).

### Fn14 Knockdown Reverses TWEAK-induced Canonical NF-κB Signal Activation and RIP1 Ubiquitination

**Figure 2:**
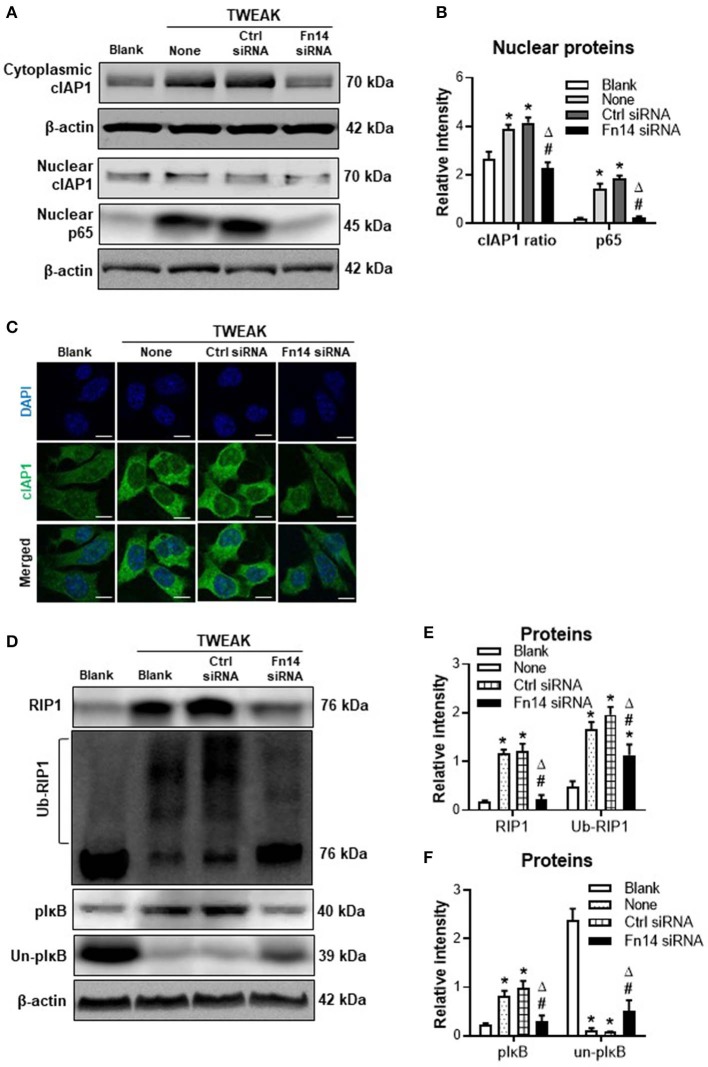
The effect of TWEAK on canonical NF-κB signals. SCC-13 cells were cultured *in vitro*, and received 48-h stimulation of TWEAK (0–1000 ng/ml). Some cells were transfected with control or Fn14 siRNA prior to TWEAK stimulation. **(A,B)** The proteins of cytoplasmic cIAP1 or nuclear cIAP1 and p65 were detected by Western blotting. **(C)** By immunofluorescence, cIAP1 was detected in cells upon TWEAK stimulation (100 ng/ml). **(D)** By Western blotting, the proteins of total RIP1, ubiquitinated RIP1 (ub-RIP1), phosphorylated IκB (pIκB), and unphosphorylated IκB (un-pIκB) were detected in cell lysates, respectively. **(E,F)** The intensities of blot bands were measured with ImageJ software. Data were obtained from three independent experiments. Representative images are shown. *p < 0.05, compared with the blank group; #*p* < 0.05, compared with the non-treated group. ^Δ^*p* < 0.05, compared with the control siRNA group.

Previous studies demonstrated that TWEAK/Fn14 interaction regulates cIAP1 effect in non-tumoral cells through activating NF-κB signals (14, 15). In this study, we revealed that TWEAK (100 ng/ml) increased nuclear expression of p65 protein in SCC-13 cells while Fn14 siRNA transfection abrogated such effect (Figures 2A,B). The cytoplasmic distribution of cIAP1 was enhanced by TWEAK but ameliorated by the transfection of Fn14 siRNA (Figures 2A–C). Moreover, transfection of Fn14 siRNA reduced the expressions of RIP1 and phosphorylated IκB (pIκB), which were regulated by TWEAK (Figures 2D–F). Accordingly, TWEAK enhanced the ubiquitination of RIP1 while Fn14 siRNA transfection attenuated such enhancement effect (Figures 2D,E). The expression of unphosphorylated IκB (un-pIκB) protein was inhibited by TWEAK but partially recovered upon Fn14 siRNA transfection (Figures 2D,F).

### cIAP1 Inhibition Abrogates TWEAK-induced Proliferation of SCC Cells

**Figure 3:**
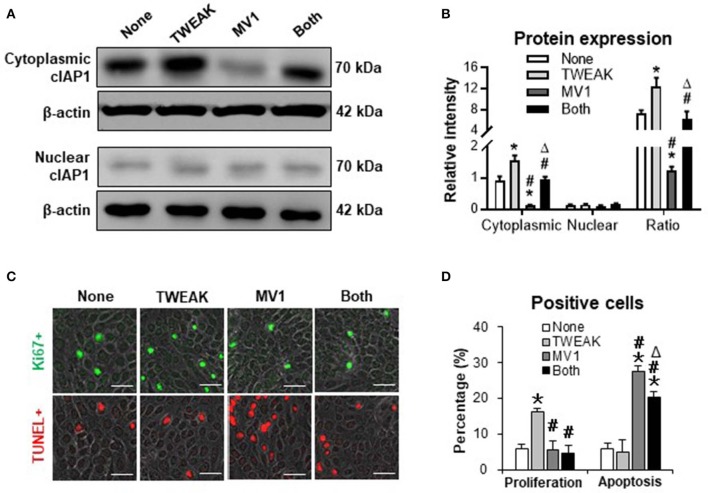
The effect of cIAP1 inhibitor on TWEAK regulation of SCC-13 cells. SCC-13 cells were cultured *in vitro*, and received 48-h stimulation of TWEAK (100 ng/ml) or plus MV1 (1 μM). **(A)** By Western blotting, the protein of cIAP1 was detected in cytoplasmic or nuclear fractions. **(B)** The intensities of Western blot bands were measured with ImageJ software. **(C)** The Ki67- (green) or TUNEL-positive (red) cells were detected by immunofluorescence. **(D)** The percentages of positive cells were calculated accordingly. Data were obtained from three independent experiments. Representative images are shown. Bar = 20 μm. *p < 0.05, compared with the non-treated group; #*p* < 0.05, compared with the TWEAK alone group. Δ*p* < 0.05, compared with the MV1 alone group.

To further investigate the mediation of cIAP1, we stimulated SCC-13 cells with TWEAK or plus an cIAP1 inhibitor. It showed that MV1 (1 μM) decreased the cytoplasmic expression of cIAP1, and also partially abrogated the promotion effect of TWEAK (100 ng/ml) on cytoplasmic distribution of cIAP1 (Figures 3A,B). In addition, MV1 significantly inhibited the proliferation of cells induced by TWEAK (Figures 3C,D). The apoptosis of cells increased upon MV1 stimulation and TWEAK attenuated such effect of MV1 (Figures 3C,D). Consistently, MV1 also reversed the enhancement effect of TWEAK on proliferation of A431 and SCC-9 cells (Figure S3).

### TWEAK/Fn14 Interaction Alters the Expression Profile of TNF-related Apoptosis-inducing Ligand (TRAIL) Receptors

**Figure 4:**
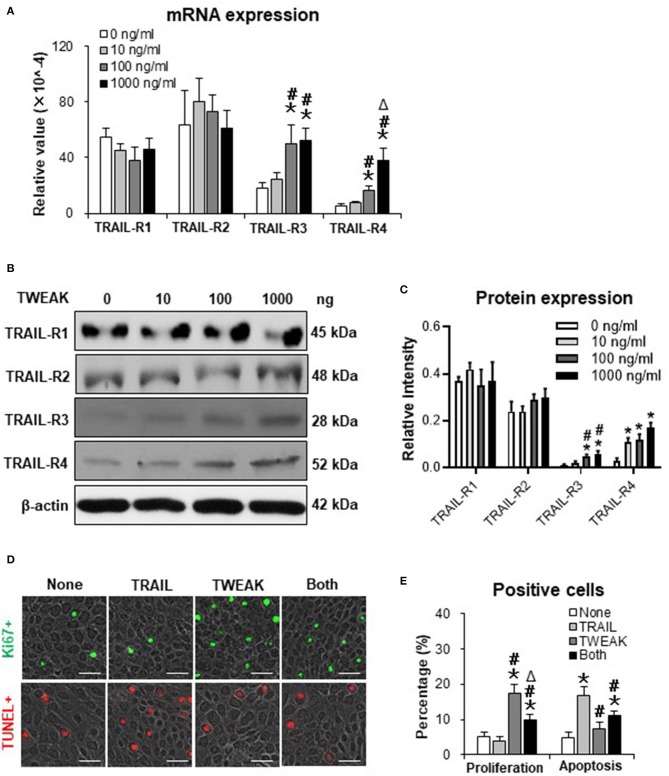
The effect of TWEAK on the expression profile of TRAIL receptors. SCC-13 cells were cultured *in vitro*, and received 48-h stimulation of TWEAK (0–1000 ng/ml). **(A)** qRT-PCR was performed for the mRNA expression levels of TRAIL receptors. **(B,C)** Western blotting was performed for the proteins of TRAIL receptors. **(D)** Cells were stimulated with TWEAK (100 ng/ml) or TRAIL (20 ng/ml) for 48 h. The Ki67- (green) or TUNEL-positive (red) cells were detected by immunofluorescence. **(E)** The percentages of positive cells were calculated accordingly. Data were obtained from three independent experiments. Representative images are shown. Bar = 20 μm. In **(A,C)**, *p < 0.05, compared with the 0 ng/ml group; #*p* < 0.05, compared with the 10 ng/ml group. ^Δ^*p* < 0.05, compared with the 100 ng/ml group. In **(E)**, *p < 0.05, compared with the non-treated group; #*p* < 0.05, compared with the TRAIL alone group. ^Δ^*p* < 0.05, compared with the TWEAK alone group.

Both TWEAK and TRAIL are members of TNF superfamily, and act differentially or cooperate in regulating cancer cells (16). Our results demonstrated that the mRNA expression levels of TRAIL receptor type 1 (TRAIL-R1) and TRAIL-R2 were not affected in SCC-13 cells by TWEAK stimulation (0 to 1000 ng/ml) while both TRAIL-R3 and TRAIL-R4 increased with the increase of TWEAK dose (Figure 4A). Accordingly, TWEAK exhibited no effect on the protein expressions of TRAIL-R1 and TRAIL-R2 but enhanced that of TRAIL-R3 and TRAIL-R4 (Figures 4B,C).

We further examined the joint effects of TWEAK and TRAIL on SCC-13 cells. It was found that TRAIL (20 ng/ml) partially abrogated the proliferative effect of TWEAK on cells (Figures 4D,E). On the other hand, TWEAK attenuated the apoptosis of cells induced by TRAIL (Figures 4D,E).

### Inhibition of TRAIL-R3/4 Abrogates the Promotion Effect of TWEAK on Cell Proliferation and cIAP1 Expression

**Figure 5:**
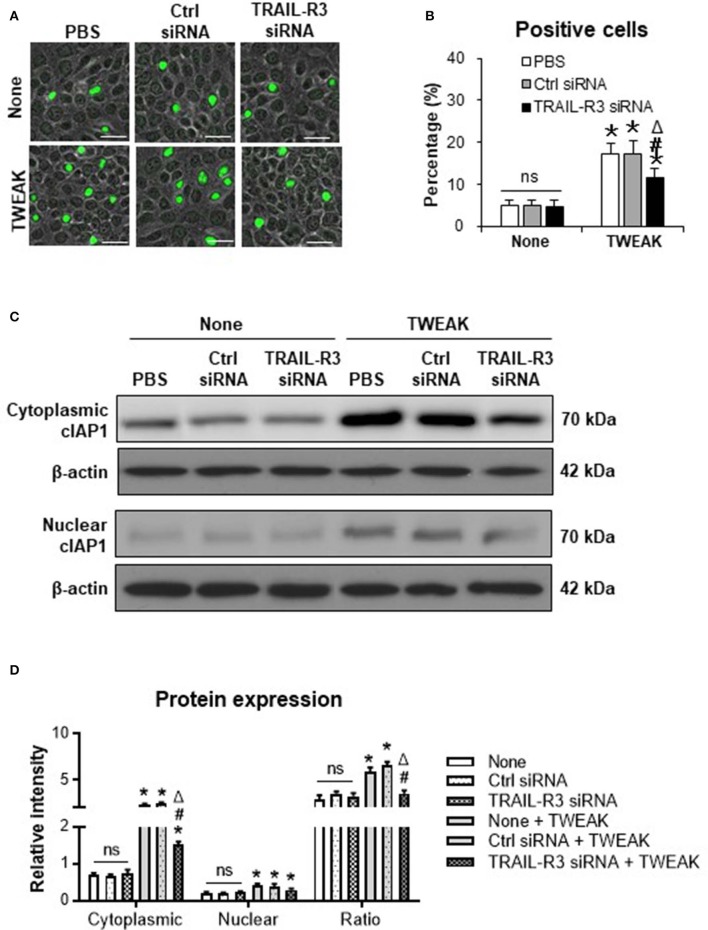
Inhibition of TRAIL-R3 abrogates the effect of TWEAK on SCC-13 cells. SCC-13 cells were cultured *in vitro*, and received 48-h stimulation of TWEAK (100 ng/ml). Some cells were pre-transfected with TRAIL-R3 or control siRNA. **(A)** The Ki67-positive (green) cells were detected by immunofluorescence. **(B)** The percentages of positive cells were calculated accordingly. **(C)** By Western blotting, the protein of cIAP1 was detected in cytoplasmic or nuclear fractions. **(D)** The intensities of blots were measured with ImageJ software. Data were obtained from three independent experiments. Representative images are shown. Bar = 20 μm. ns = not significant. *p < 0.05, compared with the non-TWEAK treated groups; #*p* < 0.05, compared with the TWEAK alone treated group. ^Δ^*p* < 0.05, compared with the TWEAK plus control siRNA treated group.

Both TRAIL-R3 and TRAIL-R4 activate antiapoptotic programs, involving the upregulation of cIAP1 in tumor cells (17, 18). In this study, we found that the transfection of TRAIL-R3 siRNA but not control siRNA partially abrogated the promotion effect of TWEAK (100 ng/ml) on proliferation of SCC-13 cells (Figures 5A,B). By Western blotting, these cells were also analyzed for the expression of cIAP1. It showed that both cytoplasmic and nuclear cIAP1 increased with the stimulation of TWEAK, and was partially attenuated upon the pre-transfection of TRAIL-R3 siRNA (Figures 5C,D). The ratio of cytoplasmic to nuclear cIAP1 also decreased upon such siRNA transfection (Figures 5C,D). Similarly, the expression of cIAP1 fluctuated with TWEAK stimulation or plus the pre-transfection of TRAIL-R4 siRNA (Figure S4).

## Discussion

**Figure 6:**
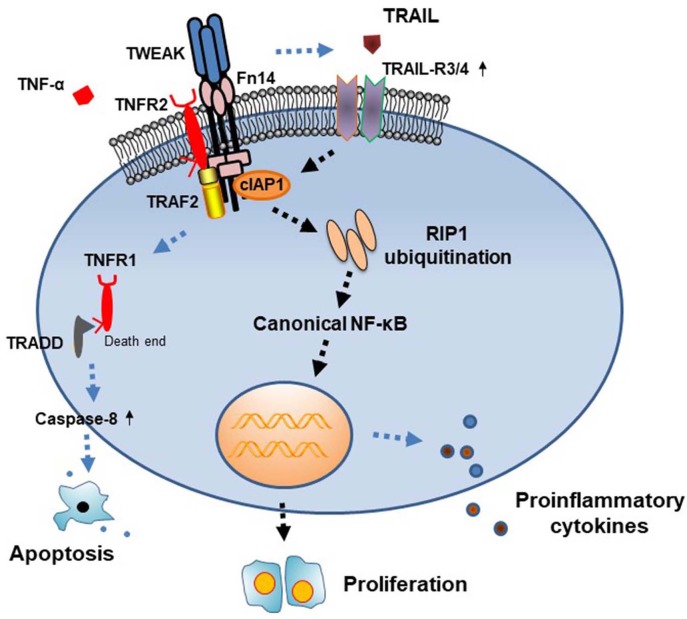
Diagram of the function of cIAP1 signals in TWEAK-induced cell proliferation. TWEAK/Fn14 interaction acts independently or cooperates with TNF-α in triggering TNFR-mediated signals. In the TNFR2-predominant cells, TWEAK upregulates the expressions of cIAP1 and TRAIL-R3/4, followed by RIP1 ubiquitination and NF-κB activation, which further trigger cell proliferation. TRADD, TNFR1-associated death domain protein.

In this study, we demonstrated that TWEAK induces cytoplasmic cIAP1 importation and RIP1 ubiquitination in SCC-13 cells, accompanied by activation of canonical NF-κB signals. Meanwhile, cIAP1 inhibition abrogates TWEAK-induced proliferation of cells. Moreover, TWEAK increases cellular expressions of TRAIL-R3 and TRAIL-R3. Furthermore, knockdown of TRAIL-R3/4 abrogates the promotion effect of TWEAK on cIAP1 expression and cell proliferation. Therefore, TWEAK/Fn14 interaction promotes the proliferation of SCC cells through activating cIAP1 signals (Figure 6).

Previous studies showed that TWEAK regulates the directions of cell fate depending on the expression profile of TNFR (8). TWEAK induces apoptosis of cells that predominantly express TNFR1 but proliferation of cells that highly express TNFR2 (12, 19–21). Moreover, TNFR2 is expressed in some tumor cells and directly promotes proliferation of cells (22). Both Fn14 and TNFR2 are highly expressed in several SCC cell lines, and inhibition of Fn14 or TNFR2 suppresses the growth of SCC xenografts in nude mice (7). Consistently, we found in this study that TNFR2 is highly expressed in SCC-13 cells. Also, TWEAK induces proliferation of SCC-13 cells in a dose-dependent manner. These results provided supportive evidences that TNFR expression determines cell fate under TWEAK/Fn14 activation.

TNFR-associated factor-2 (TRAF2) binds to cIAP1 and recruits it to the cytoplasmic domain of several members of the TNFR superfamily, activating the NF-κB signals (23). cIAP1 has the capacity to bind and ubiquitylate several signaling intermediates including TRAF2 and RIP1 (24). Cytoplasmic recruitment of cIAP1 is a prerequisite for NF-κB activation, and triggers cytokine signaling (25). Previously, we found that TWEAK increases the proliferation of and cytoplasmic import of cIAP1 in TNFR2-overexpressing keratinocytes (8). In this study, we further demonstrated that TWEAK induces cytoplasmic cIAP1 importation and RIP1 ubiquitination in SCC cells. In fact, RIP1 ubiquitination correlates positively with NF-κB signaling activity and acts as an oncogenic driver in cancer cells (26, 27). Upregulation of Fn14 increases Ras GTPase activity and TRAF2 expression in human papillomavirus E6/E7-transfected keratinocytes, which harbor tumorigenicity and prefer to proliferation under TWEAK stimulation (12). Therefore, TWEAK induces proliferation of SCC cells involving the TRAF2-cIAP1-RIP1 axis-mediated NF-κB signaling.

Both TWEAK and TRAIL are members of TNF superfamily, and act differentially or cooperate in regulating various cells (16, 28, 29). There are four types of TRAIL-Rs, including TRAIL-R1, TRAIL-R2, TRAIL-R3, and TRAIL-R4. The activation of TRAIL-R1 or TRAIL-R2 signaling induces apoptosis of cancer cells. However, TRAIL-R3 and TRAIL-R4 have a TRAIL-binding domain but lack cytoplasmic domains required for apoptosis activation. They have a fifth TRAIL binding protein named osteoprotegerin, which is able to sequester TRAIL, thus suppressing TRAIL-R1/2-dependent apoptosis (30). We found that TWEAK exerts no effect on the expressions of TRAIL-R1 and TRAIL-R2 but dose-dependently upregulates the expressions of TRAIL-R3 and TRAIL-R4 in SCC cells. Moreover, TWEAK attenuates the apoptosis of SCC cells induced by TRAIL. Inhibition of TRAIL-R3/4 abrogates the promotion effect of TWEAK on cell proliferation and cytoplasmic cIAP1 expression. Our results are consistent with the facts that both TRAIL-R3 and TRAIL-R4 activate antiapoptotic programs involving the upregulation of cIAP1 in tumor cells (17, 18). The precise mechanism underlying the TWEAK regulation of TRAIL-Rs needs further investigations.

Targeting cIAP1 is a novel strategy for suppressing oncogenic activity and chemotherapy drug resistance in tumors (31, 32). MV1 is a cIAP1-recognizing moiety and specifically depletes endogenous cIAP1/cIAP2/XIAP in cells (33). MV1 can be conjugated to other small molecules without alteration of biological effect (34). In this study, we observed that MV1 decreases the cytoplasmic expression of cIAP1 in SCC cells, and also partially abrogated the promotion effect of TWEAK on cIAP1 distribution and cell proliferation. This finding confirms that cIAP1 inhibition abrogates TWEAK-induced proliferation of SCC cells. Considering the high expression of Fn14 in tumor cells and the specific affinity of TWEAK to Fn14, the conjugate of TWEAK to an Fn14 or cIAP1 inhibitor (e.g., MV1) might be a promising strategy for the treatment of malignant tumors.

Taken together, TWEAK/Fn14 interaction activates cIAP1 signals and enhances proliferation of SCC cells, involving the TRAIL-R expression profile and downstream NF-κB signals. Specific inhibition of cIAP1 abrogates the promotion effect of TWEAK on cell proliferation. These findings suggest therapeutic strategies for suppressing proliferation of cancer cells. Future studies should be focused on the design of TWEAK-conjugated cIAP1 inhibitors as well as the verification of *in vivo* or *in vitro* effect.

## Data Availability Statement

All datasets generated for this study are included in the article/Supplementary Material.

## Author Contributions

LL and CC participated in the design of the study, and performed most experimental work. GH, XW, and JL carried out some experiments. ZY discussed the experimental data and contributed to the interpretation of results. WZ and YX conceived and designed the study and prepared the manuscript. All the authors read and approved the final manuscript.

### Conflict of Interest

The authors declare that the research was conducted in the absence of any commercial or financial relationships that could be construed as a potential conflict of interest.

## Abbreviations

cIAP1: cellular inhibitor of apoptosis 1
Fn14: fibroblast growth factor-inducible 14
NF-κB: nuclear factor kappa B
qRT-PCR: quantitative real-time polymerase chain reaction
RIP1: receptor-interacting serine/threonine-protein kinase 1
SCC: squamous cell carcinoma
TNF: tumor necrosis factor
TNFR: TNF receptor
TRAIL: TNF-related apoptosis-inducing ligand
TRAIL-R: TRAIL receptor
TWEAK: TNF-like weak inducer of apoptosis.
