# Supplementary material for: TWEAK Promotes the Proliferation of Squamous Cell Carcinoma Cells Through Activating cIAP1 Signals
Source: Front Oncol. 2020 Apr 15;10:439. doi: 10.3389/fonc.2020.00439 (PMC7174721; doi:10.3389/fonc.2020.00439)
Supplement: Supplementary file 1 [file Presentation_1.pdf]

Table S1. Primer sets used for sequencing expression genes

Gene	Primer (5’-3’)
Human Fn14	F: CTCTGAGCCTGACCTTCGTG<br>R: GGGGGCACATTGTCACTGGA
Human TRAIL-R1	F: GAGAGTTGTGTCCACCAGGA<br>R: GGTGCAGGGACTTCTCTCTT
Human TRAIL-R2	F: GGACAGAAGCTCACAACGAC<br>R: ACATGTTGACACCTGTTGGC
Human TRAIL-R3	F:GGGTGTGGATTACACCAACG<br>R: CTAGGGCACCTGCTACACTT
Human TRAIL-R4	F: CCAGGTCTCTGAGCAGGAAA<br>R: CTTCAGCTTCTGCCTGTTCC
Human GAPDH	F: GCACCGTCAAGGCTGAGAAC<br>R: TGGTGAAGACGCCAGTGGA

Abbreviations: Fn14, fibroblast growth factor-inducible 14; TRAIL-R, tumor necrosis factor-related apoptosis-inducing ligand receptor; GAPDH, glyceraldehyde 3-phosphate dehydrogenase; F: forward; R: reverse.

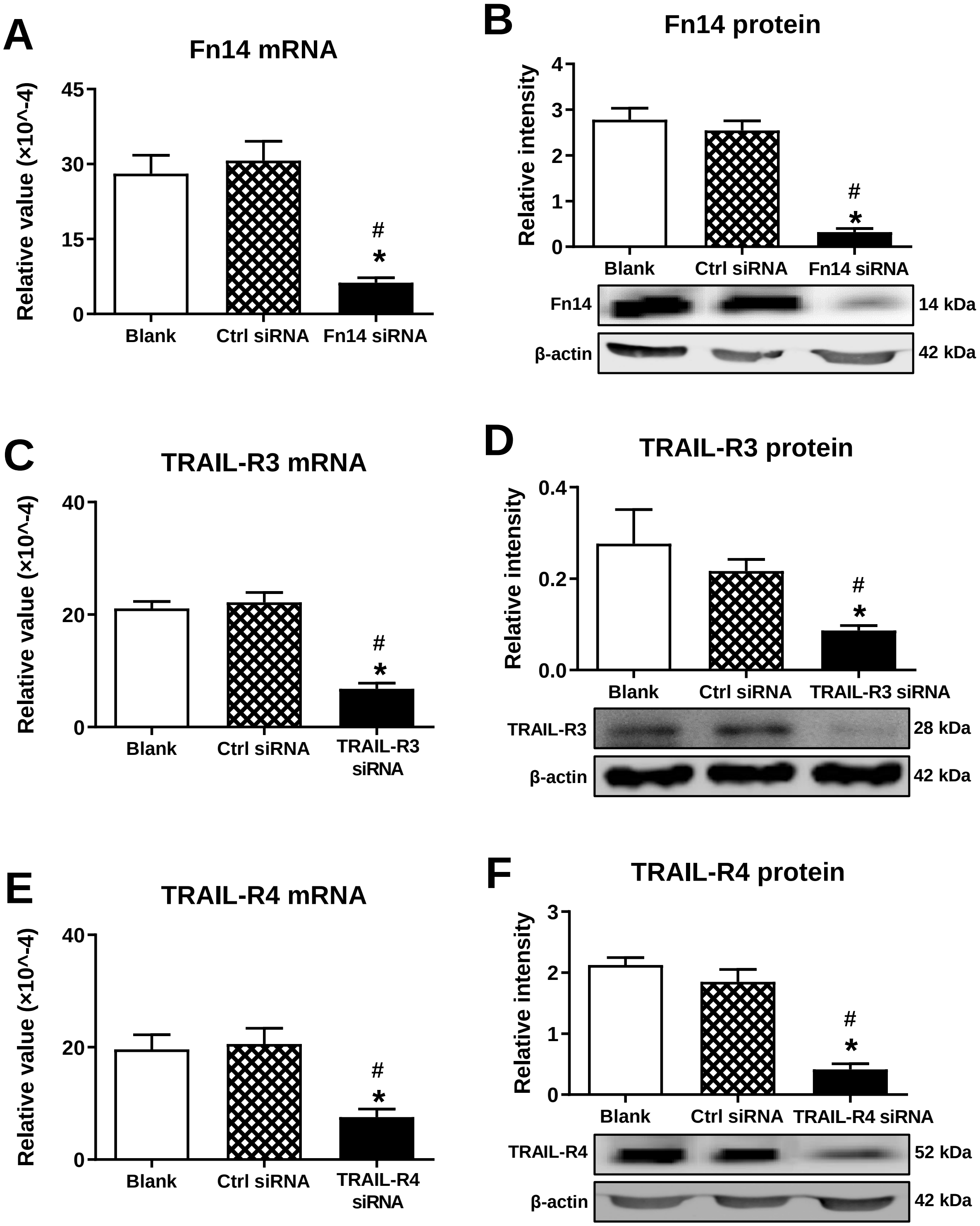

Figure S1. The efficiencies of siRNA transfection. SCC-13 cells were cultured *in vitro*. Some cells were transfected with control or target siRNA. (**A**) The mRNA expression level of Fn14 was determined by qRT-PCR. (**B**) The protein of Fn14 was detected by Western blotting. The intensities of blot bands were measured with ImageJ software. (**C, D**) The mRNA and protein expression levels of TRAIL-R3 were determined by qRT-PCR and Western blotting, respectively. (**E, F**) The mRNA and protein expression levels of TRAIL-R4 were determined accordingly. Data were obtained from three independent experiments. Representative images are shown. *$p < 0.05$, compared with the blank group; #$p < 0.05$, compared with the control siRNA group.

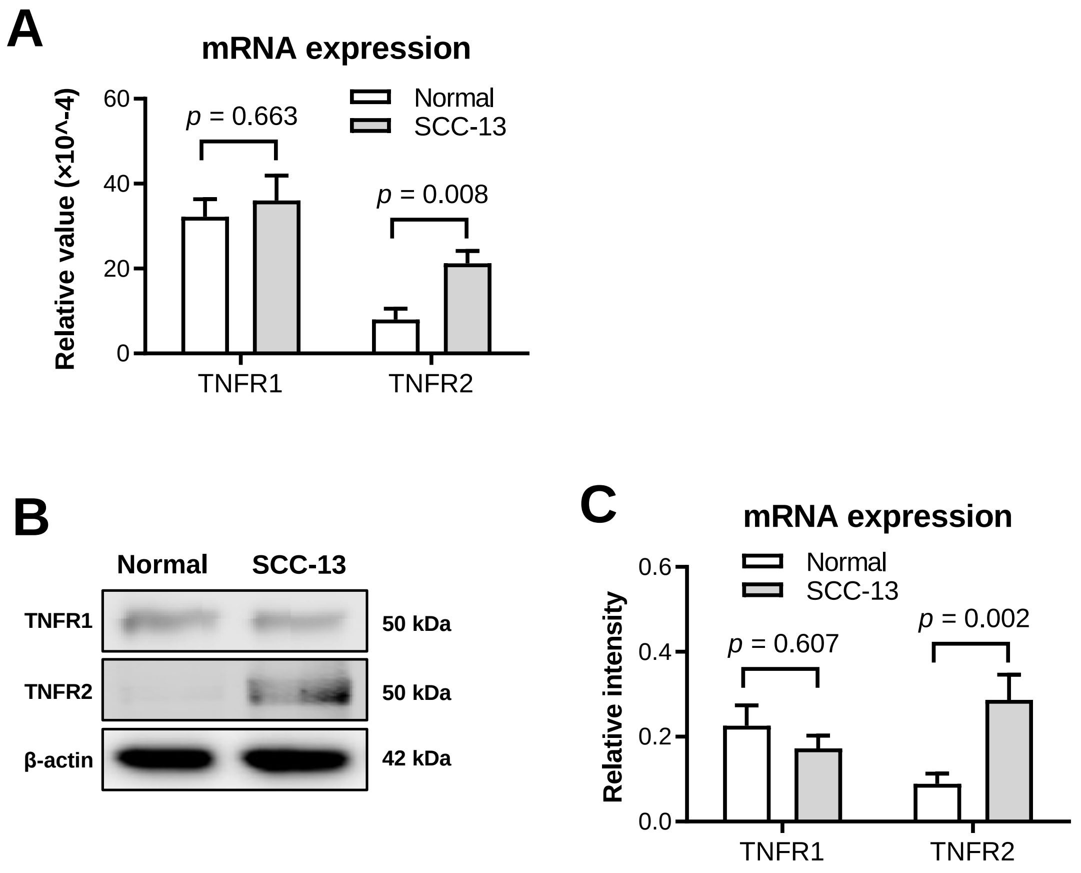

Figure S2. TNFR2 is highly expressed in SCC-13 cells. Both human primary keratinocytes and SCC-13 cells were cultured *in vitro*. (**A**) By qRT-PCR, the mRNA expression levels of TNFR1 and TNFR2 were determined in cells. (**B**) By Western blotting, the proteins were detected in cell lysates. (**C**) The intensities of blots were measured with ImageJ software. Data were obtained from three independent experiments. Representative images are shown.

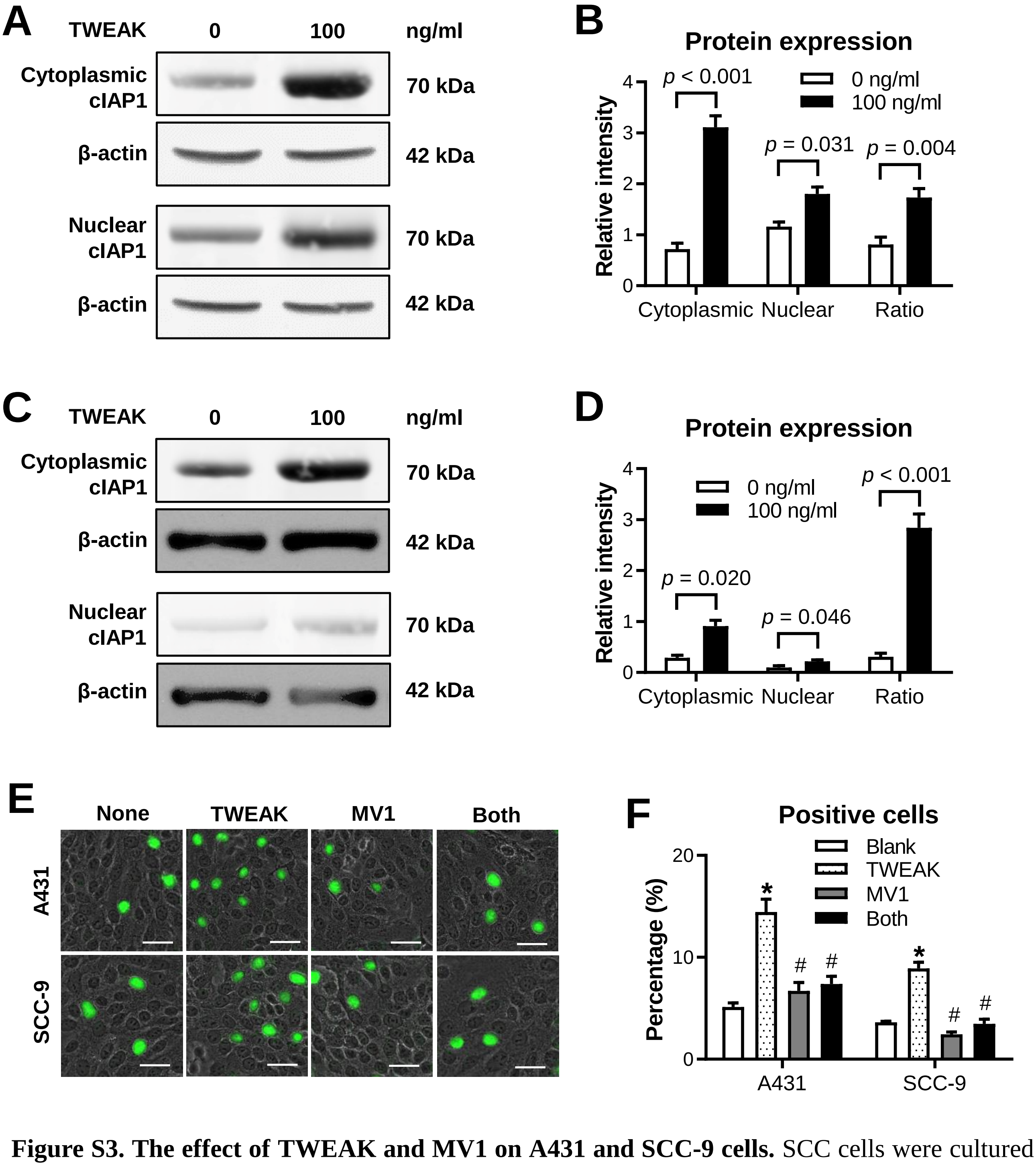

Figure S3. The effect of TWEAK and MV1 on A431 and SCC-9 cells. SCC cells were cultured *in vitro*, and received 48-h stimulation of TWEAK (100 ng/ml) or MV1 (1 μM). (**A**, **B**) By Western blotting, the protein of cIAP1 was detected in cytoplasmic or nuclear fractions from A431 cells. The intensities of blot bands were measured with ImageJ software. (**C**, **D**) Similarly, the protein of cIAP1 was detected in cytoplasmic or nuclear fractions from SCC-9 cells. (**E**, **F**) The Ki67-positive (green) cells were detected by immunofluorescence. The percentages of positive cells were calculated. Data were obtained from three independent experiments. Representative images are shown. Bar = 20 μm. *$p < 0.05$, compared with the blank group; #$p < 0.05$, compared with the TWEAK alone group.

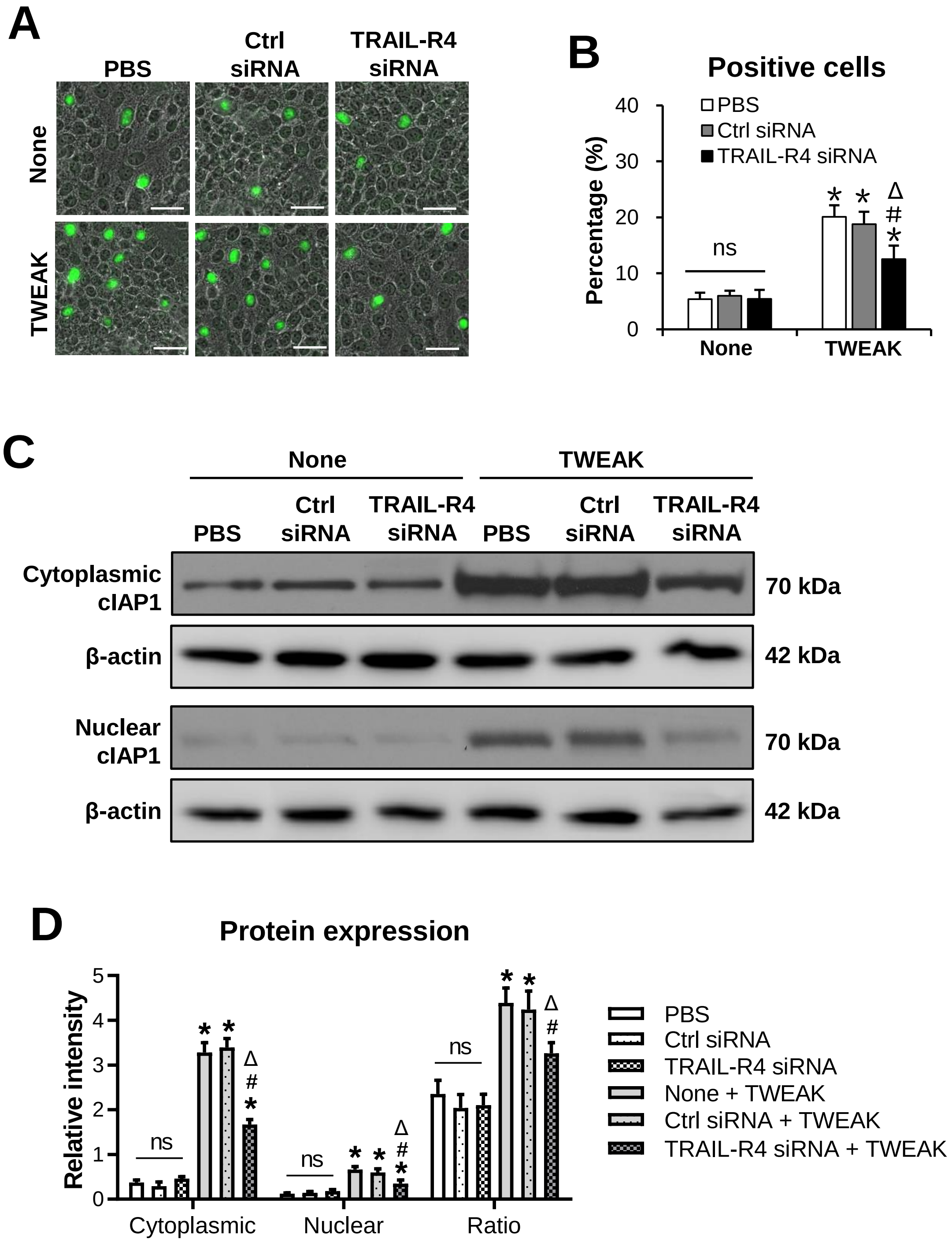

Figure S4. Inhibition of TRAIL-R4 abrogates the effect of TWEAK on SCC-13 cells. SCC-13 cells were cultured *in vitro*, and received 48-h stimulation of TWEAK (100 ng/ml). Some cells were pre-transfected with TRAIL-R4 or control siRNA. (**A**) The Ki67-positive (green) cells were detected by immunofluorescence. (**B**) The percentages of positive cells were calculated accordingly. (**C**) By Western blotting, the protein of cIAP1 was detected in cytoplasmic or nuclear fractions. (**D**) The intensities of blots were measured with ImageJ software. Data were obtained from three independent experiments. Representative images are shown. Bar = 20 μm. ns = not significant. *$p < 0.05$, compared with the non-TWEAK treated groups; #$p < 0.05$, compared with the TWEAK alone treated group. Δ$p < 0.05$, compared with the TWEAK plus control siRNA treated group.
